# Undergraduate medical student exposure to OMFS, a future workforce concern—a narrative review of the literature

**DOI:** 10.1308/rcsann.2025.0038

**Published:** 2025-10-17

**Authors:** I Perwaiz, A Mackenzie, U Rehman, MS Sarwar, PA Brennan

**Affiliations:** ^1^ Bradford Teaching Hospitals, UK; ^2^ Charing Cross Hospital, UK; ^3^ UCL Division of Surgery and Interventional Sciences, UK; ^4^ Queen Alexandra Hospital, UK

**Keywords:** Oral surgery, Undergraduate medical education, Maxillofacial surgery

## Abstract

**Introduction:**

Oral and maxillofacial surgery (OMFS) is a surgical specialty dealing with a broad spectrum of pathology affecting the head and neck. Despite a rising burden of head and neck conditions, exposure to OMFS in the undergraduate medical curriculum remains limited. This has consequences for medical undergraduates’ competence and confidence in managing related conditions and their recruitment into this specialty.

**Methods:**

A literature search was conducted in September 2024 on Pubmed, Dynamed, DARE, EMBASE, Cochrane and BMJ electronic databases for articles published between 1970 and 2024. The review was registered with PROSPERO (CRD42024556491).

**Results:**

Ten studies involving 2,499 participants were included. Only 33.1% of students had experience in OMFS, with variations in exposure type ranging from formal teaching sessions to clinical placements. Most exposures were brief, typically less than a week, and often did not extend beyond basic lectures or shadowing. A notable proportion of students expressed a desire for more comprehensive training in OMFS. Only 12.8–37% of participants were considering a career in OMFS. Low interest rates were attributed to dual qualification requirements, cost and duration of training.

**Conclusions:**

The findings highlight a major gap in OMFS education delivered by medical schools. This is concerning given that OMFS in the UK is struggling to fill higher surgical training posts. If interest in the profession remains low, we may encounter a recruitment and workforce crisis. Future strategies must focus on meaningful opportunities to increase OMFS experience in the medical curriculum while addressing other barriers to recruitment and retention.

## Introduction

Oral and maxillofacial surgery (OMFS) focuses on the diagnosis and surgical treatment of conditions affecting the head and neck.^1^ The burden of disease has evolved due to advances in medical technology, demographic shifts, and lifestyle variations.^2,3^ As a result, the specialty manages a wide range of both simple and increasingly complex presentations. This includes management of oral pathologies, traumatic facial injuries, head and neck cancer (HNC) and reconstruction of congenital defects.^4,5^

An increasing lifespan attracts a rising burden of disease, and OMFS is not unacquainted with this. There are approximately 12,000 new HNC diagnoses in the UK each year.^6^ Approximately 30% of HNCs are diagnosed when the disease is localised; survival rates at five years are two to four times lower in those who receive a late diagnosis.^7^ Prior research demonstrates deficits in undergraduate medical teaching of OMFS with minimal exposure to facial trauma. Therefore, medical students do not feel confident in assessing or managing any traumatic facial injuries, including simple lacerations.^8,9^

The undergraduate dental curriculum focuses extensively on the study of the head and neck region. It covers various elements of anatomy, surgery, head and neck pathology and facial trauma. Our prior research has demonstrated that, whereas medical students are keen to develop their knowledge and competence in OMFS, the UK undergraduate medical curriculum does not place the same emphasis on OMFS compared with the dental counterpart.^9,10^

This is problematic because most patients who present with an oral or maxillofacial condition will initially present to a non-specialist practitioner. This paper will aim to explore the current exposure to OMFS in the undergraduate medical curriculum as well as attitudes, motivators and barriers towards pursuing a career in the specialty among undergraduate medical students.

## Methods

The review utilised the International Prospective Register of Systematic Reviews (PROSPERO) from the University of York’s Centre for Reviews and Dissemination to minimise bias. The review was registered with PROSPERO (CRD42024556491).

### Literature search

A literature search was conducted in September 2024 on Pubmed, Dynamed, DARE, EMBASE, Cochrane and British Medical Journal (BMJ) electronic databases for articles published between 1970 and 2024. The following search parameters were used to retrieve the relevant articles: ‘oral and maxillofacial surgery’, ‘OMFS’, ‘exposure’, ‘undergraduate’, ‘undergraduate exposure’, ‘undergraduate attitude’, ‘medical school’, ‘facial trauma’, ‘head and neck’, ‘facial anatomy’, ‘head and neck anatomy’, ‘skin cancer’, ‘facial skin cancer’, ‘cleft’, ‘temporomandibular joint’ and ‘salivary gland’.

Only original research studies published between 1970 and 2024 were considered. The following study types were reviewed: randomised controlled trials, prospective cohort studies, retrospective cohort studies, case studies and case series. Each item was initially screened for eligibility and inclusion based on its title and abstract. Subsequently, the full papers were reviewed before inclusion.

### Inclusion

This article included all studies focusing on undergraduate medical students, exposure to and attitudes towards OMFS, as well as studies examining undergraduate education and exposure to OMFS subspecialties. These subspecialties include facial trauma, head and neck surgery, cleft lip and palate, and salivary gland surgery. Studies were included from all geographical locations.

### Exclusion

For this article, studies focusing on undergraduate exposure and attitudes towards surgical specialties other than OMFS were excluded. Studies focusing on dental students or dental curriculum did not meet the inclusion criteria. Studies focusing on postgraduate trainees were excluded.

### Data extraction

The population comprised undergraduate medical students across all year groups in the UK. The intervention was exposure to OMFS at the undergraduate level. No comparators were used. Outcomes measured included undergraduate exposure to OMFS, nature of exposure and career interest.

### Risk of bias and quality assessment

Each study was assessed for selection bias, comparability and outcome reporting risks using the Newcastle – Ottawa Scale for cohort studies. The results were then converted into scores using the Agency for Healthcare Research and Quality criteria.

## Results

**Figure 1:**
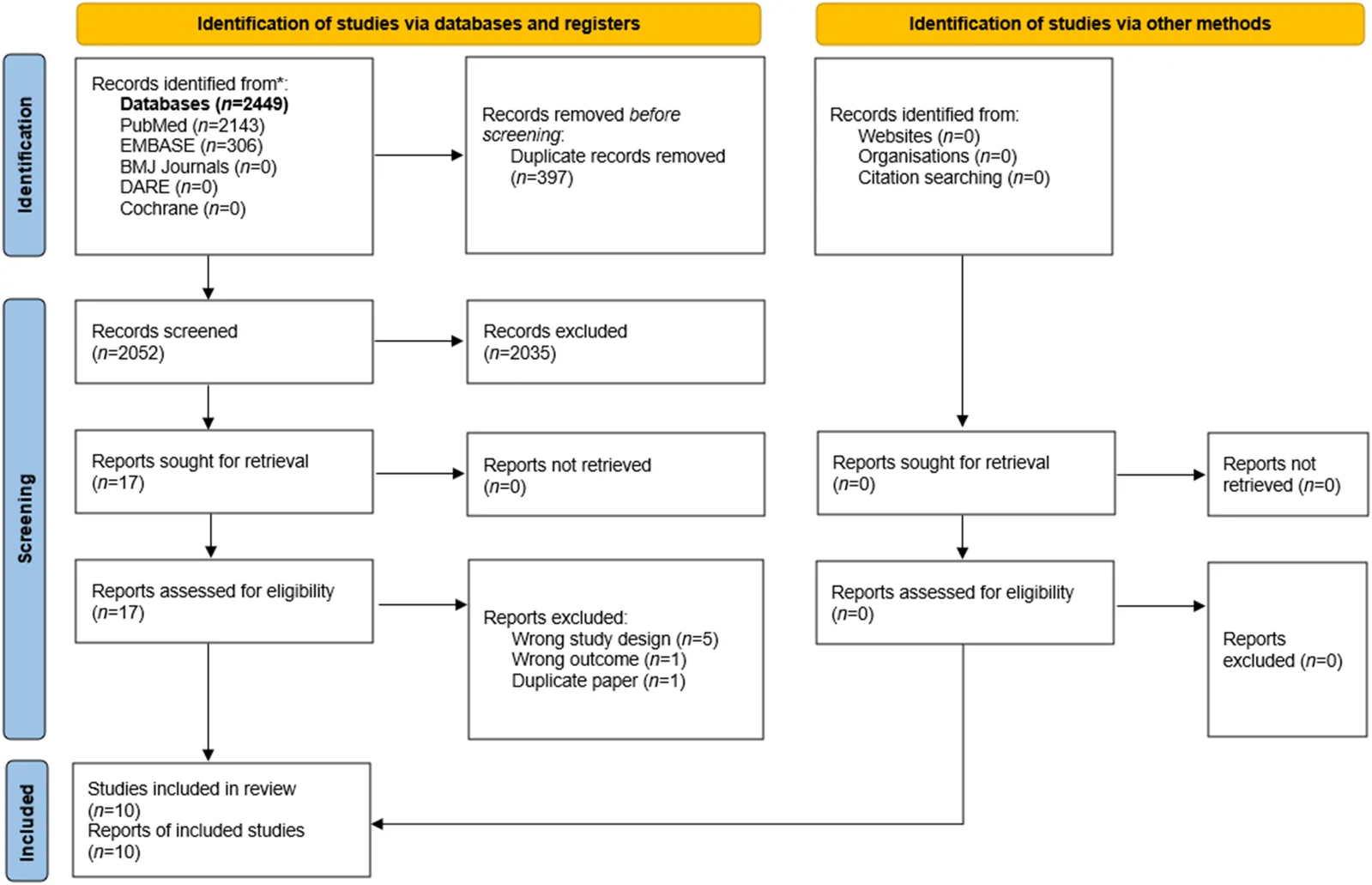
PRISMA flowchart

The full numbers of articles screened, assessed for eligibility and included in this review, with reasons for exclusion, are presented in the PRISMA flow diagram (Figure 1). A literature search using the key search terms as defined earlier identified a total of 2,449 results; 397 duplicates were removed. Grey literature searches identified zero results. A total of 2,035 abstracts were screened. Full-text review was performed for 17 articles. In total, ten articles fulfilled inclusion criteria and were included in our study. Study characteristics can be seen in Table 1.

**Table 1:** Study characteristics

Title	Author and year	Country	Number of students	Universities	Students with undergraduate exposure to OMFS	Type of exposure	Length of exposure	More exposure required?	Understanding of OMFS	Career interest	Deterrents and motivators	Training pathway awareness
An evaluation of oral and maxillofacial surgery in UK medical schools^11^	Kalia *et al* 2012	UK	155	Not mentioned	18.9% (*n*=29)	Timetabled, 11% Not mentioned	Not mentioned	Y, 73.5% (*n*=114)	Not mentioned	Not mentioned	Not mentioned	Not mentioned
Awareness of oral and maxillofacial surgery as a speciality and potential career pathway amongst UK medical undergraduates^12^	Goodson *et al* 2013	UK	253	2, London	27.7% (*n*=70)	Clinical placement, 13.0% (*n*=33) Teaching in OMFS, −11.5% (*n*=29) Work experience prior to medical school, 4.0% (*n*=10)	Clinical placement: <1 week, 5.5% (*n*=14) >1 week but <1 month, 2.8% (*n*=7) ≥1 month, 4.7% (*n*=12)	Y, 76.7% (*n*=194) wanted more guidance on career	Not mentioned	Y, 18.0% (*n*=35/194)	Dual qualification, 41.2% (*n*=80)	Y, 27.7% (*n*=70)
Oral and maxillofacial surgery in medical schools in the United Kingdom^13^	Mahalingam *et al* 2015	UK	186	5–2 in London, other locations not mentioned	24% (*n*=45)	Own initiative, (*n*=37), own initiative, taster sessions, contacting OMFS departments, special study modules and electives Timetabled, (*n*=8)	Not mentioned	Y, 89% (*n*=166)	Not mentioned	Y, 21% (*n*=39)	Length of training pathway, 20% (*n*=38) Dual qualification, 18% (*n*=33)	Not mentioned
Medical students’ understanding of oral and maxillofacial surgery: an Irish perspective^14^	Kielty *et al* 2017	Ireland	443	2, Cork and RCSI	14.9% (*n*=56/374)	10.2%, 38/374, theoretical teaching only 4.8%, 18/374, some degree of clinical teaching	4, clinical teaching >5 days	Y, 64% (*n*=212/329)	Not mentioned	Y, 9% (*n*=34/329 (9%)	Not mentioned	Y, 11/367 (3%)
Knowledge of final-year medical students about oral and maxillofacial surgery: a two-centre study^15^	Hamid *et al* 2018	UK	200	2, Oxford and St George’s	Lectures, 36% (*n*=72) Rotation, 11% (*n*=22) Tutorials, 12% (*n*=24)	Lectures, 36% (*n*=72) Rotation, 11% (*n*=22) Tutorials, 12% (*n*=24)	1 lecture or more, 36% (*n*=72) 1 rotation week or more, 11% (*n*=22)	Not mentioned	56.3% (*n*=563/1000)	Not mentioned	Not mentioned	Not mentioned
A multi-site cross-sectional survey exploring medical undergraduate knowledge of oral and maxillofacial surgery^16^	Harris *et al* 2019	UK	299	16	22.8% (*n*=69)	Incidental exposure, 14.1% (*n*=42) Teaching, 7.7% (23) Clinical placement, 5.7% (*n*=17) Student selected component, 2.35% (*n*=7)	Not mentioned	Not mentioned	Referrals made to OMFS: Facial bone fractures, (93.9%) Congenital facial deformities (82.4%) Cleft lip and palate surgery (79.0%) Orthodontic provision (15.3%) Toothache (10.2%) Denture provision (11.9%). (13.9%), tracheostomy	Not mentioned	Not mentioned	Significantly higher awareness among OMFS-exposed students compared with OMFS naïve
Familiarity and awareness of facial cosmetic and oral maxillofacial surgery among dental and medical undergraduate students: multicentre cross-sectional study^17^	Binyamin *et al* 2020	Saudi Arabia	452	7, (AlFarabi College, Batterjee Medical College, Ibn Sina National College for Medical Studies, Jeddah University, King Abdulaziz University, King Saud Bin AbdulAziz University for Health Sciences, Umm Al-Qura University) in the Makkah region of Saudi Arabia.	Nonspecific ‘surgical rotations’, 35% (*n*=158.2) Information about OMFS on rotation, 23.7% (*n*=107)	Not mentioned	Not mentioned	Not mentioned	Knowledge regarding OMFS, 74.8%	Y, 12.8%	Not mentioned	Not mentioned
Facial trauma education within two English medical schools^9^	Rehman *et al* 2022	UK	2, locations not mentioned	33% (*n*=79)	Clinical placement teaching, 29% (*n*=69) Medical school surgical society events 4% (*n*=10)	1-2 hours, 33% (*n*=78) >2 hours, 0.4% (*n*=1)	Not mentioned	Referrals to OMFS for facial fracture (cumulative): 31% (*n*=295) Breakdown: Zygomatic, 37% (*n*=87) Mandibular, 74% (*n*=179) Orbital floor, 10% (*n*=24) Nasal, 2% (*n*=5)	Not mentioned	Not mentioned		
Attitude of clinical medical students to Oral and Maxillofacial Surgery as a career: a perspective from two English medical schools^9^	Rehman *et al* 2022	UK	198	2, Locations not mentioned	38.90% (*n*=77)	10.1% (*n*=20), student selected components (SSCs) 2.0% (*n*=4) -quality improvement projects/audits related to OMFS	1-7 days, 24.8% (*n*=49) 1-4 weeks, 12.1% (*n*=24) 4–8 weeks, 2.0% (*n*=4)	69.7% (*n*=138)	Not mentioned	Y, 37.0% (*n*=72)	Motivators: 34.3% (*n*=68) responded diversity in workload 26.3% (*n*=52) stated potential for private practice 17.2% (*n*=34) responded academic interest 4.0% stating prestige (*n*=8) 5.0% (*n*=10), personal experiences with OMFS *n*=6, medical electives *n*=2 and reading about OMFS *n*=2. Deterrents: Dual qualification - 44.4% (*n*=88) Length of training, 20.2% (*n*=40) Alternative similar specialties 16.1% (*n*=32) Limited exposure, 10.1% (*n*=20) Not interested in surgery, 2.5% (*n*=5) were not interested in surgery Work-life balance, 2.0% (*n*=4) Financial implications, 2.0% (*n*=4) Competition, 1.5% (*n*=3) Family plans, 1.0% (*n*=2)	Y, 30.3% (*n*=60)
Exposure and awareness of oral and maxillofacial surgery for first degree medical undergraduates in the United Kingdom^18^	Jaibaji *et al* 2023	UK	76	15	9.2% (*n*=7), placement 15.8% (*n*=12), teaching	9.2% (*n*=7), placement15.8% (*n*=12), teaching	9.2% (*n*=7) - Mean placement length of 4 days	82.9% (*n*=63)	Not mentioned	Not mentioned	Dual qualification - 28.9% (*n*=22) Financial expenses, 17.1% (*n*=13)	Not mentioned

OMFS = oral and maxillofacial surgery; Y = yes

### Study characteristics

The selected studies were published between 2012 and 2023, and they included 2,499 participants across multiple institutions. Studies were conducted primarily in the UK (*n*=8); other nations included Saudi Arabia (*n*=1) and Ireland (*n*=1). The main themes identified were exposure to OMFS, career interest, and recruitment and retention issues as well as the understanding of OMFS.

### Exposure to OMFS

All studies (*n*=10) commented on student exposure to OMFS. Exposure rates to OMFS varied across studies and institutions. Overall, 33.1% (*n*=827) of participants had some form of OMFS exposure. The OMFS exposure rate between institutions ranged from 9.2% to 38.9%.

Participant experience with OMFS was predominantly in the form of teaching or lectures (*n*=310), followed by clinical placements (*n*=148), and self-directed exposure to student-selected components (*n*=64). Six studies commented on the length of exposure.^9,12,14,15,18^ Reporting of this varied across studies (range, one hour to eight weeks). Exposure was generally short in nature, with only 69 students reporting OMFS exposure of one week or more. Six studies explored participant perceptions on whether greater experience in OMFS is required. The percentage of students in need of increased exposure and guidance in OMFS ranged between 64% and 89%.^9,11–14^

#### Career interest

Five studies commented on interest in pursuing a career in OMFS.^9,12–14,17^ Participant interest was low, with only 237 students showing a positive interest in pursuing OMFS as a career. The interest ranged from 12.8% to 37% between studies. One study by Rehman *et al* noted motivators to pursue OMFS as a career,^9^ the most common being diversity in workload (34.4%, *n*=68), potential for private practice (26.3%, *n*=52) and academic interest (17.2%, *n*=34). Substantial deterrents to the pursuit of OMFS as a career were also highlighted across four studies (*n*=713).^9,12,13,18^ Deterrents included the requirement for dual qualifications (31.3%, *n*=223), long length of the training pathway (10.9%, *n*=78), desire to pursue allied specialties (4.49%, *n*=32) and limited exposure (2.81%, *n*=20).

#### Understanding of OMFS

Three studies commented on the understanding of OMFS and referral patterns. The ability to apply OMFS knowledge varied, with some studies considering appropriate clinical referrals made by students to OMFS services as satisfactory. 56.3% of students demonstrated adequate basic knowledge by making correct referrals to OMFS (Hamid *et al*).^15^ Rehman *et al* discovered that some students could make referrals for facial bone fractures (31%, *n*=295)^9^; however, this was significantly lower than the findings reported in another study performed by Harris *et al*,^16^ where 93.9% correctly referred facial bone fractures. Notably, Harris *et al* reported that students who had some exposure to OMFS demonstrated significantly higher awareness and familiarity with the field compared with their unexposed counterparts.^16^

Four articles commented on awareness and understanding of the OMFS training pathway (*n*=818) and found that, on average, 17.2% (*n*=141) students were aware of the training pathway (range 3–30.3%).^9,12,14,16,17^

## Discussion

### Experience in OMFS

Participant experience in OMFS varied considerably between institutions in both the nature of exposure and duration, ranging from 9.2% to 38.9%. Experience and interest go hand in hand, and several studies have highlighted that inadequate educational experiences and limited exposure at the undergraduate level can influence career interest and understanding. The potential gap in medical education can be exploited to increase interest in OMFS as a specialty.^19–22^ It is widely accepted that a person’s decision to pursue a surgical specialty is generally established in undergraduate years.^23–25^ Medical undergraduates are therefore less likely to pursue a career in OMFS given the low exposure rate.

The types of OMFS exposure included clinical placements, theoretical teaching, and student-selected components, the majority of which lasted less than one week. Most students (64–89%) reported insufficient exposure, suggesting that a single week of experience is insufficient to develop a basic understanding and make a well-informed decision to pursue OMFS as a potential career. Shadowing has consistently been identified as a critical influence on students’ decisions to pursue surgery.^26^

Traditionally, OMFS has been a dental-first specialty; however, the British Association of Oral and Maxillofacial Surgeons (BAOMS) has found that a growing number of trainees have medicine as their first qualification. Despite the rising trend of medicine-first graduates pursuing OMFS, the specialty remains underrepresented at the undergraduate medical level in comparison with dentistry. The literature indicates that dental students have significantly higher exposure to OMFS (71.8%) during their primary dental qualification, whereas only 33.1% of medical students reported exposure to OMFS, as highlighted in this review. This imbalance underscores the need for adequate exposure to OMFS in both dental and medical curricula.^27,28^

OMFS is highly relevant not only to those pursuing the specialty but also to all foundation doctors rotating through emergency medicine, where facial trauma is encountered frequently. Without fundamental knowledge, critical complications such as retrobulbar haemorrhages may be missed or managed with delays, leading to adverse outcomes. Furthermore, with increasing difficulties in accessing dental care, issues such as oral pain and lesions are increasingly presenting to General Practice and emergency departments. Awareness and exposure to OMFS oral cancer clinics at the undergraduate level can help establish a foundational understanding of oral assessments, ensuring safe and appropriate referrals.

There is an urgent need for medical schools to re-evaluate the undergraduate curriculum and incorporate a range of opportunities for OMFS exposure. This may include integration into ENT rotations or the introduction of dedicated head and neck rotations where allied specialties such as ENT, OMFS and Plastic Surgery receive equal representation. Such measures are essential to ensure comprehensive coverage of the specialty and to produce safe and competent doctors.

### Understanding of OMFS

Many students lacked an insight into the scope of the specialty. A significant proportion of participants reported the need for more guidance, in-depth exposure to OMFS and lack of understanding as significant barriers to entry.^29^ Harris *et al* reported that students who had some exposure to OMFS demonstrated significantly greater awareness compared with their unexperienced counterparts.^16^

Medical students report head and neck anatomy as a difficult subject to grasp, and sound learning requires teaching and repeated exposure.^30,31^ Disparities exist in understanding that are directly related to the duration of experience and type of educational content provided during training. Kielty *et al* reported that only a small percentage of students received clinical teaching, which may contribute to the broader misunderstanding of the specialty’s scope and practice.^14^ On the other hand, Hamid *et al* demonstrated that only 56.3% of participants possessed basic OMFS knowledge.^15^ This is concerning given the crucial role OMFS plays in managing complex facial injuries, congenital deformities and malignancy.^15,31^ This strongly emphasises the need for educational reform to improve OMFS curricula in medical schools, as insufficient exposure is detrimental to understanding the scope of the specialty and implications for patient care.

### Career interest, recruitment and attrition

The limited exposure negatively impacts knowledge and career interest. OMFS interest as a career varied from 12.8% to 37% between studies. In their study, Rehman *et al* reported that 37.0% of students expressed a desire to pursue OMFS, influenced by the diversity of the workload and the potential for private practice.^9^ However, deterrents such as dual qualification requirements and length of training negatively impacted the interest. These findings are echoed by Goodson *et al* and Jaibaji *et al*, who found only 18.0% and 28.9% of students, respectively, consider a career in OMFS.^12,18^ This under-representation in career preferences can perpetuate the cycle of limited exposure and ultimately give rise to potential recruitment concerns for OMFS.^32^ Magennis *et al* raised concerns about the reduction in the number of consultants stemming from recruitment issues, and evolving job plans among other factors to include pension pressures.^33^ The resolutions proposed to maintain capacity were highlighted as increasing the number of consultant posts and training numbers while making efforts to actively retain senior surgeons in OMFS.^33^

In the UK, it is not uncommon for OMFS training posts to remain vacant, even after several rounds of recruitment.^34^ A lack of OMFS cover in these regions would give rise to a postcode lottery scenario where access to OMFS care is no longer universal for patients. The additional workload would be redistributed across other specialties such as ENT or plastic surgery. The net effect may increase the likelihood of indefinite removal of the OMFS training post as the other specialties assume the work to meet the needs of the local population.

Other barriers to OMFS pursuit as a specialty include financial cost and dual qualification requirements, which are mirrored in the literature, with one study citing 51% of students being deterred by the length of training and 31% by financial burdens.^29,35^ The cost of OMFS training is estimated to amount to £113,000 if medicine is completed as a first degree, whereas costs to train in other surgical specialties range between £22,000 and £26,000.

This can be further exacerbated by the loss of income and pension contributions during the second degree and the high number of courses required by OMFS in comparison with other specialties.^36^

OMFS as a specialty faces significant recruitment and retention challenges in current times. This is attributable to the demanding nature of the training, cost and the dual qualification requirements. The lengthy albeit rigorous pathway may result in stress, burnout and, ultimately, attrition among trainees.^37^ A combination of these factors without mitigation and improving wellbeing may further render OMFS less appealing to train in. If this issue is not addressed, there may be an insufficient number of OMFS consultants in the future to manage the growing burden of head and neck disease. This could ultimately lead to the fragmentation of the specialty, with its workload being absorbed by allied fields such as ENT or Plastic Surgery.

### Transferable skills from OMFS

Students’ ability to apply their knowledge in clinical settings was reviewed. Harris *et al* highlighted that a considerable percentage of students made correct referrals to OMFS for conditions such as facial bone fractures (93.9%), congenital deformities (82.4%) and cleft palate (79%), thus indicating a limited but functional understanding of when to consult OMFS in clinical practice.^16^ Hence, small interventions might be effective in teaching the practical aspects of the specialty. However, these findings contrast work done by Rehman *et al*, where only 31% of students referred facial fractures to OMFS.^9^ This is likely due to varied educational experiences, which can lead to a lack of confidence among medical graduates when assessing facial trauma and potentially leading to important pathology being missed. Medical students will rotate through Emergency Departments during their Foundation Years, where they are expected to assess and manage facial trauma. This highlights the urgent need for improved and comprehensive basic training in OMFS in undergraduate medical education for safe and effective management of common presentations as future clinicians.

## Future directions

We have established that educational interventions are associated with greater awareness, understanding and recruitment toward OMFS.^16^ Adequate representation of OMFS in the medical and dental curricula is essential, not only to prepare prospective trainees for the specialty, but also to equip future trainees in general specialties such as emergency medicine or general practice to provide appropriate care and ensure prompt referral. Proactive approaches including integrated clinical rotations, interactive workshops and case-based learning sessions can enhance students’ familiarity with the specialty, making it more accessible and appealing.^38,39^ Specific curricular enhancements could include incorporation of facial trauma to emergency medicine rotations and ensuring that ENT rotations incorporate OMFS training, particularly in oral cancer, which is an increasing issue for our population.^2,9^

Streamlining the training pathways and integrating dual-degree programs would reduce the duration by combining medical and dental training and adopting a modular competency-based training pathway as opposed to a time-based format.^40^ The deficit in OMFS exposure can be addressed by making it more accessible through the use of simulation tools and augmented reality, making training more practical. Future strategies must focus on making the profession more appealing to first-degree medical students. This will positively reflect enhanced recruitment and retention in OMFS and a thriving workforce that can cater to wide demographic and population needs in the UK.

## Conclusions

Our findings suggest a critical need for interventions in medical curricula to improve both the depth and breadth of OMFS exposure. Increasing and standardising OMFS experience in the undergraduate medical curriculum would improve both understanding of and interest in OMFS. In turn, this could increase the appeal of the specialty and thus the number of students considering it as a future career. Addressing barriers to entry and demystifying the training pathway are also key areas of focus to enable this. Greater experience and interest in OMFS would in turn lead to improved patient outcomes and help avoid a potential future workforce crisis borne out of a lack of exposure to the specialty.

## Limitations

Variability in study designs and methodologies across the included studies makes it challenging to compare results directly or generalise findings across different educational settings. Most of the studies are from the UK; therefore, generalisability to other settings is limited. There are inconsistencies in how data are reported, particularly regarding the types of exposure and the metrics used to assess students’ understanding and interest in OMFS, which prevents a comprehensive analysis. In addition, some data from earlier studies might not reflect current educational practices or student experiences due to evolution over time.

The review also focuses primarily on exposure and awareness without an extensive examination of the effectiveness of various educational interventions in enhancing understanding or interest, which could have provided more objective and actionable insights. Addressing these limitations in future research could involve broader geographical sampling, standardised reporting and a mix of qualitative and quantitative research methods to capture a global picture of the educational landscape surrounding OMFS in the UK.
